# Newborn hearing screening protocol in tuscany region

**DOI:** 10.1186/s13052-017-0397-1

**Published:** 2017-09-20

**Authors:** Stefano Berrettini, Paolo Ghirri, Francesco Lazzerini, Giovanni Lenzi, Francesca Forli

**Affiliations:** 1 Otolaryngology Audiology and Phoniatric Unit, University of Pisa, Pisa, Italy; 2 Mother and Child Department, Neonatology Unit and Section of Neonatal Endocrinology and Dysmorphology, University Hospital of Pisa, Pisa, Italy; 3 Paediatrician, National Coordinator of FIMP Audiology section, Grosseto, Italy

**Keywords:** Screening, Hearing loss, Newborn, CMV, Paediatric

## Abstract

**Background:**

Newborn hearing screening has to be considered the first step of a program for the identification, diagnosis, treatment and habilitation/rehabilitation of children with hearing impairment.

**Main Part:**

In Tuscany Region of Italy, the universal newborn hearing screening is mandatory since november 2007. The first guidelines for the execution of the screening have been released in June 2008; then many other Italian regions partially or totally adopted these guidelines. On the basis of the experience from 2008 and according to the recent evidences in the scientific literature, a new screening protocol was released in Tuscany region. The new protocol is an evolution of the previous one. Some issues reported in the previous protocol and in the Joint Committee on Infant Hearing statement published in 2007 were revised, such as the risk factors for auditory neuropathy and for late onset, progressive or acquired hearing loss. The new updated guidelines were submitted to the Sanitary Regional Council and then they have been approved in August 2016. The updated screening protocol is mainly aimed to identify newborns with a congenital moderate-to-profound hearing loss, but it also provides indications for the audiological follow-up of children with risk’s factor for progressive or late onset hearing loss; further it provides indications for the audiological surveillance of children at risk for acquired hearing impairment. Then, in the new guidelines the role of the family paediatrician in the newborn hearing screening and audiological follow-up and surveillance is underscored. Finally the new guidelines provide indications for the treatment with hearing aids and cochlear implant, in accordance with the recent Italian Health Technology Assessment (HTA) guidelines.

**Conclusions:**

In the paper we report the modality of execution of the universal newborn hearing screening in the Tuscany Region, according to the recently updated protocol. The main features of the protocol and the critical issues are discussed.

**Electronic supplementary material:**

The online version of this article (10.1186/s13052-017-0397-1) contains supplementary material, which is available to authorized users.

## Background

The universal newborn hearing screening (NHS) is aimed to identify babies with moderate to profound hearing impairment present at birth (unilateral or bilateral) [1, 2].

NHS has to be considered as a first step of a program for the identification, diagnosis, treatment and habilitation/rehabilitation of children with hearing impairment [1].

Tuscany is a region in central Italy with an area of about 23,000 km^2^ and a population of about 3.8 million inhabitants (2016).

Totally, there are 26 childbirth points in Tuscany: 25 are public structures and 1 is in a private clinic. In 2015 488.000 babies were born in Italy, of which 27.500 in Tuscany (5,64%).

In Tuscany Region of Italy, the first guidelines for the execution of the Universal Newborn Hearing Screening (UNHS) have been released in June 2008; then many other Italian regions partially or totally adopted these guidelines. Furthermore, in the 2017 “essential health care levels” (livelli essenziali di assistenza, LEA, in Italian language) document, the NHS is reported to be mandatory for each Italian region.

In 2011 a report by the Regional Agency for Health on NHS execution in Tuscany attested that 100% of the Tuscanian childbirth clinics executed the UNHS and that more than 98% of the neonates born in Tuscany underwent the procedure [2].

On the basis of the experience from 2008 and according to the recent evidences in the scientific literature, a new screening protocol was released in Tuscany region. The new protocol was based on the previous one and on the Joint Committee on Infant Hearing (JCIH) statement published in October 2007. In the JCIH statement, indeed, there is a list of all auditory risk factors marked with different symbols, in order to point out the indicators associated with an higher risk (e.g. received extracorporeal membrane oxygenation or positivity for CMV infection). All infants with indicator for hearing loss are reported to be referred for an audiological assessment at least once by 24–30 months of age; children with risk factors associated with an higher risk for delayed-onset hearing loss have to substain more frequent audiological assessments [1]. These aspects were revised and modified and the new updated guidelines were submitted to the Sanitary Regional Council and then they have been approved in August 2016.

The updated screening protocol is mainly aimed to identify newborns with a congenital unilateral or bilateral, moderate-to-profound hearing loss, but it is also provides indications for the audiological follow-up of children with risk’s factor for progressive or late onset hearing loss; further it provides indications for the audiological surveillance of children at risk for acquired hearing impairment. Then, in the new guidelines we underlined the role of the family paediatrician in the newborn hearing screening and audiological follow-up and surveillance.

Finally the new guidelines provide indications for the treatment with hearing aids (HA) and cochlear implant (CI), in accordance with the recent Italian Health Technology Assessment (HTA) guidelines [3].

In the following sessions we report the modality of execution of the UNHS in the Tuscany Region in Italy, according to the recently updated protocol.

## Methods

In Tuscany, the centres that are involved in the NHS and in the early diagnosis and treatment of hearing impairment in childhood are divided in 3 levels, depending on facilities and personnel available in the childbirth and in the audiologic clinic. The modalities of screening execution are adjusted on the bases of each centre’s resources.

### Level I centre

- the childbirth unit has the last generation’s facilities for measurement of Transient Evoked Oto-Acoustic Emissions (TEOAE) only. The test is executed by the paediatrics nurse, paediatrician, and/or the audiometrist, audiologist or ENT specialist, after an appropriate training.

### Level II centre

- the childbirth unit has the last generation’s facilities for measurement of TEOAE and the equipment for automatic auditory brainstem responses (AABR) or clinical auditory brainstem responses (ABR). The test is executed by the audiometrist, the audiologist or, eventually, from the ENT specialist, the paediatrician, the paediatrics nurse, after an appropriate training.

### Level III centre

- the structure has the last generation facilities for TEOAE and AABR in childbirth clinic; further the structure is provided with an audiological ward including the equipment for clinical ABR with threshold definition, clinical TEOAE, Distortion Produced Oto-Acoustic Emissions (DPOAE), impedance audiometry and behavioural audiometry; furthermore the structure has experienced personnel in the early diagnosis of hearing loss, in the rehabilitation of children with HA or CI, and it has a suitable arrangement for the execution of aetiological investigations (infectious disease specialist, geneticists, neuroradiologists, ex.)

The screening procedure should be always executed within the discharge of the baby from the childbirth unit, except for few complex situations (e.g. newborn voluntary early discharge). In those situations an appointment for the screening procedures is given at the discharge, according to the audiological centre’s personnel. The childbirth centre repeats the test within 2 weeks from the birth of the babies for verify the *refer* cases; a dedicated structure for collect babies with refer *results* can be arranged.

In the paper published by our group in 2011 the 1,04% of neonates born in the Neonatal Unit of Santa Chiara Hospital in Pisa, underwent a further audiologic assessment after been defined as *refer* at NHS. In the 0,42% of all the children underwent the NHS, has been made a final diagnosis of hearing impairment [2].

Each hospital must identify for each childbirth unit a person in charge of screening’s procedures and databases; this professional would be a paediatrician or another specialist (audiologist and/or ENT specialist), and he/she would involve nurses and/or audiometrists in the execution of the screening’s tests.

We suggest that each childbirth centre selects qualified personnel for the execution of the tests (paediatrician, audiometrist, paediatrics nurse, audiologist, ENT specialist). Generic operators has to be avoided.

All the childbirth centres with an high number of births per year, the centres with a neonatal intensive care unit (NICU), the II and the III level centres should have a continuous connection with the Audiology/ENT unit and dedicated audiomestrists. Further, specific training courses can be arranged for the operators in each local area.

The access at screening’s procedure for children born in private hospitals in Tuscany region has been established, as well as the procedures for children born at home.

Furthermore, babies born in other Italian regions or abroad and adopted children that did not attend the screening procedures, must undergo hearing screening (or other audiologic tests adequate for the age of the subject) in the audiologic centre, within one month from the birth or from the taken in charge from the paediatrician.

Is it necessary to differentiate the execution’s modality of the UNHS in newborns with or without risk’s factors for auditory neuropathy (AN). In Table 1 we report risk’s factors for AN.

**Table 1:** Risk factors for AN

1.	Hospitalization in NICU for more than 5 days
2.	Positive familiar anamnesis for infantile permanent hearing impairment
3.	Positive familiar anamnesis for neurodegenerative disorders, as Hunter syndrome, sensitive-motor neuropathies, Friedreich’s atassia, Chacot-Marie-Thoot syndrome.

#### Screening on newborns without risk’s factors for auditory neuropathy (Fig. 1)

TEOAE in childbirth centres are measured by the responsible personnel within the discharge of the baby from the childbirth unit generally after 24 h of life. The TEOAE should be measured during the spontaneous sleep of the baby after the nutrition, possibly in silence. If the TEOAEs are present (*pass*) on both ears the procedure is concluded. If in one or both the ears no TEOAEs (*refer*) are measured, another measurement in both ears [1] is repeated after some hours, but within the discharge of the baby. If the TEOAE are bilaterally present at the second measurement (*pass*), the procedure is concluded. If TEOAEs are absent in one or both ears and if personnel and facilities are disposable for the measurement of AABR (level II centre), an AABR measurement is done within the discharge of the baby from the childbirth unit; if AABR are normal (*pass*) the procedure is concluded. In fact, AABR (with stimulus level set at 45 dB HL) has got an high specificity, providing a very small number of false positive cases. TEAOEs, indeed, is a test more sensible then the AABR, but it can present an higher number of false positive cases. For that reason we decided to consider *pass* at screening a newborn with *pass* AABR and *refer* TEAOEs, but we also considered necessary that those babies has to be closely followed by the paediatrician.

**Fig. 1:**
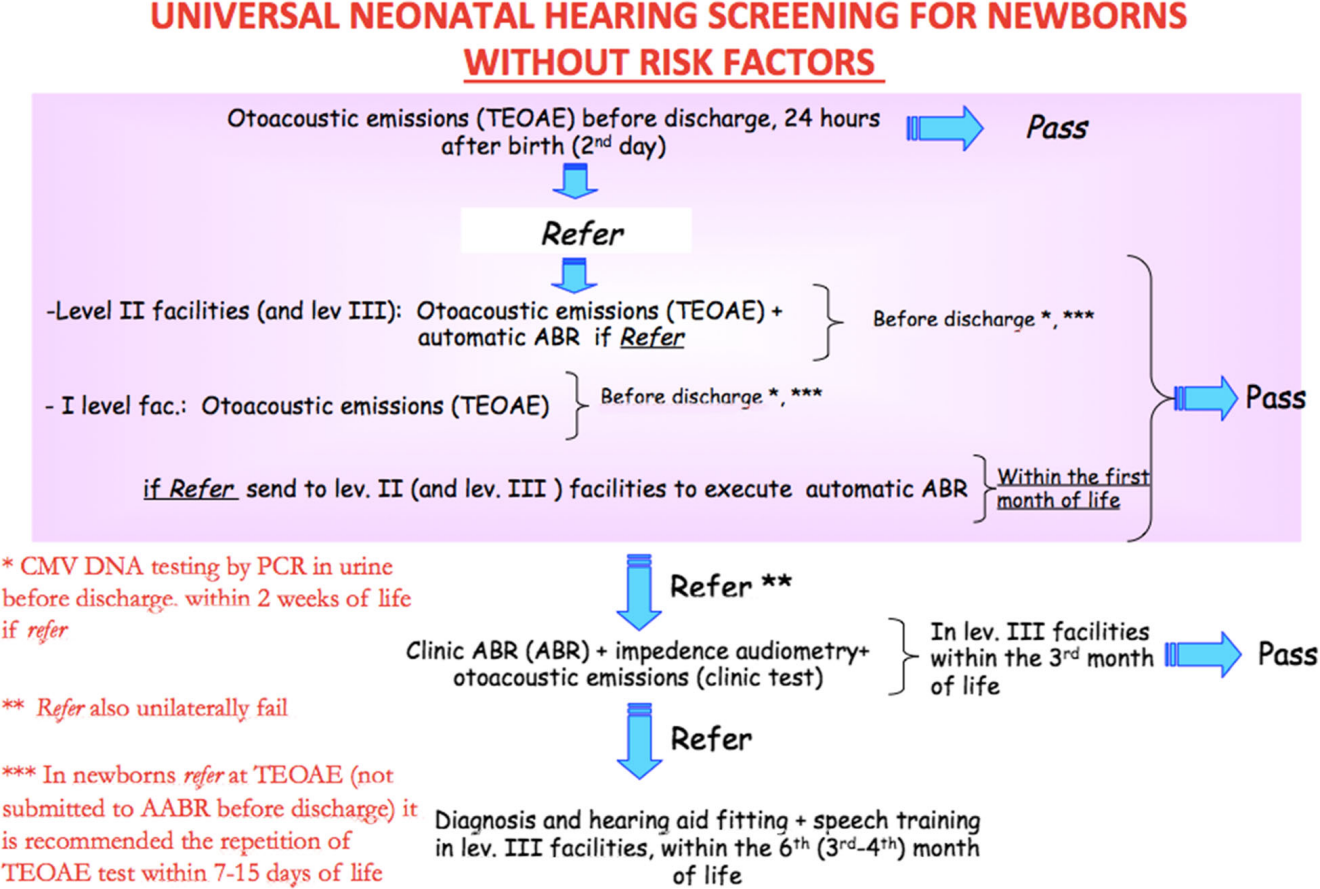
Universal neonatal hearing screening for newborns without risk factors. Legend: flowchart for the neonatal hearing screening in newborns without risk factors for auditory neuropathy

In newborns resulted *refer* at screening procedures with TEAOE that have not executed AABR before the discharge, a repetition of TEAOE in both ears within 7–15 days is suggested.

Cases that still result *refer* at second TEOAE measurement are addressed to II or III level centres for the II level screening’s procedure with the execution of ABR (automatics or clinical) within a month from the birth.

Babies resulted refer at II level screening’s procedure (TEOAE + AABR) are addressed within III month from the birth, to III level centres for a diagnostic-audiologic complete assessment (clinical ABR with threshold evaluation, impedance audiometry, stapedial reflexes + TEOAE, ex.). Then, further diagnostic sessions, hearing aids fitting and rehabilitation procedures can be performed, and within the 6th month from the birth of the baby a therapeutic and rehabilitative treatment has to be assessed. In newborns with severe-profound bilateral hearing loss, an as-early-as-possible (3rd - 4th month) hearing aids fitting and speech therapy is desirable to reduce as much as possible the time of hearing deprivation.

All the newborns resulted *refer* at I level screening with TEOAE must be submitted to CMV DNA research in urine by PCR within the 15th day from the birth.

At the discharge from the childbirth centre, the person in charge of the screening procedure or its collaborators must:

1. report UNHS results on baby’s paediatrics booklet
2. report on the register of childbirth centre the result, the execution’s modalities of the screening and the category of the baby: with or without risk’s factors for auditory neuropathy, with or without risk’s factors for progressive or late onset hearing impairment
3. report the screening’s result in an online secured database
4. inform the parents and the paediatrician of hearing screening’s results. Further, if the baby result *refer* UNHS, the childbirth centre’s personnel must inform to the parents, verbally and with an informative paper about the instruction for the II level evaluative tests
5. having previously contacted the responsible audiologic centre, the personnel must inform the parents about the appointment for the II or III level evaluative tests (an informatics booking system between the audiologic centre and the childbirths centre is suggested)
6. the II level evaluative tests have to be executed within a month from the discharge from the childbirth centre and the audiological diagnosis has to be accomplished within 3 months from the discharge from the childbirth centre

The booking system and the database permit to reduce lost to follow-up cases. In fact for each newborn resulted *refer* at NHS, a new retest in the audiologic centre is scheduled before the discharge. Further the responsible personnel constantly check the database, verifying that all the *refer* cases execute the audiologic evaluation.

#### Screening on newborns with risk’s factors for auditory neuropathy (Fig. 2)

A distinction between the execution of screening protocol in newborns with and without risk’s factors for auditory neuropathy is needed. So, newborns with the characteristics reported in Table 1 must undergo the screening both with TEOAE and AABR, in order to identify possible cases of auditory neuropathy. It is well known that AN has an higher incidence in the categories of babies reported in Table 1 and it is not identified with TEOAE (that may results present in those patients).

**Fig. 2:**
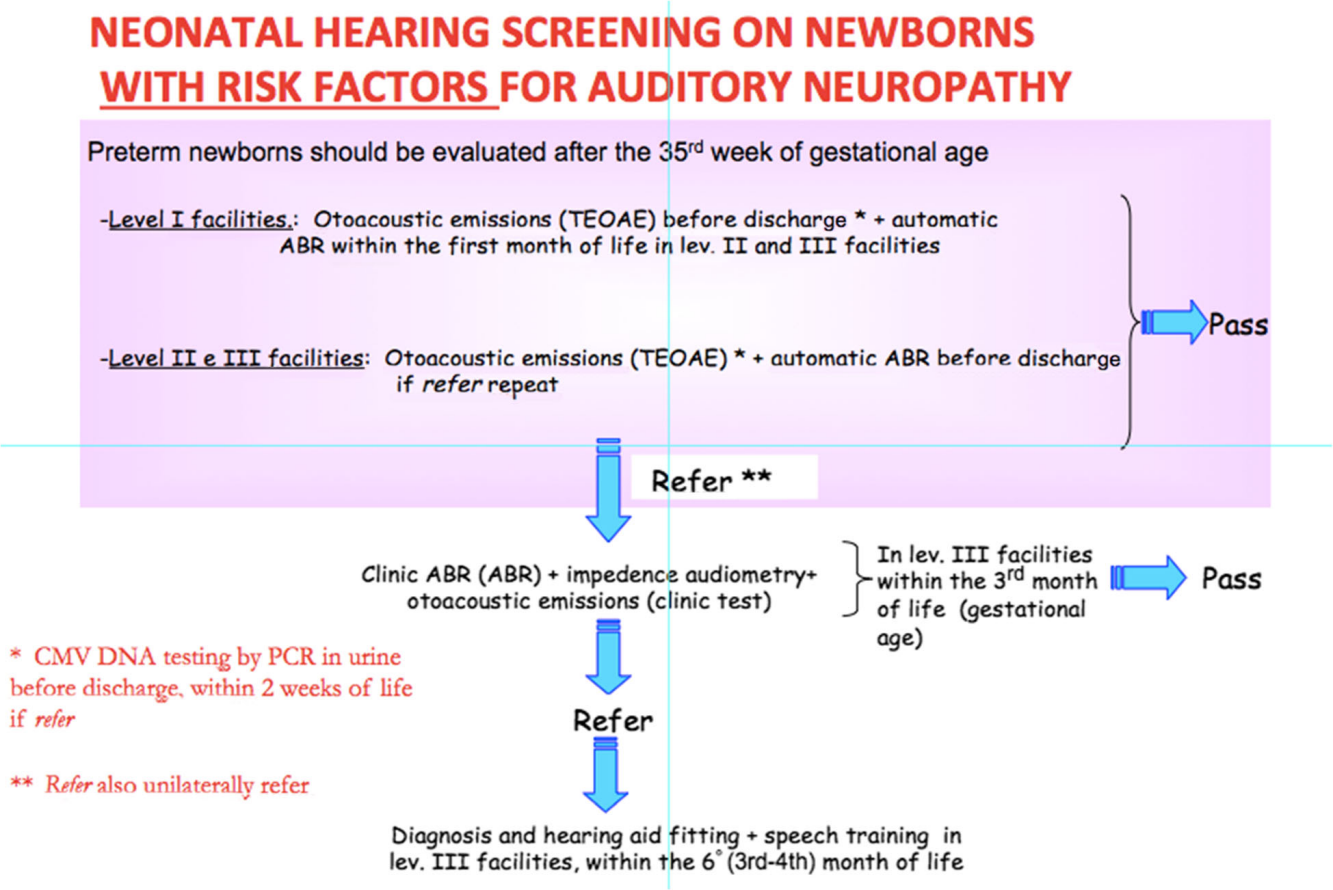
Universal neonatal hearing screening for newborns with risk factors. Legend: flowchart for the neonatal hearing screening in newborns with risk factors for auditory neuropathy

All the newborns that show the condition reported in Table 1 must undergo a specific diagnostic protocol.

If the newborn is preterm, the screening’s procedure has to be executed at the end of the 35th gestational week or after, but within the discharge, in order to reduce the incidence of false positive; before the 35th week, indeed, the execution of TEOAE may be difficult for the limited dimensions of the external auditory canal and the AABR’s responses may be not revealed for an immaturity of the central hearing pathways.

Babies belonging at the categories reported in Table 1 have to execute the TEAOE and AABR measurement before the discharge from the childbirth centre or within a month (II level centres). If the result is *refer*, is suggested to execute the tests at least 2 times before the discharge. For this procedure is strongly suggested a collaboration with skilled personnel (in particularly with audiometrist).

Newborns resulted *refer* at TEOAE must execute, within the 15th day from the birth, the research of CMV DNA by PCR in urine.

Babies resulted *refer* for one or both ears are addressed to the III level centre for an audiological evaluation.

At the discharge, the responsible personnel for the screening procedure or its collaborators must follow the guidelines reported in the previous paragraph at point 1, 2, 3, 4, 5, 6.

Babies included in risk categories that was born in I level centre, must undergo AABR in II or III level centre to complete the screening’s procedure within a month from the birth.

#### Surveillance on children with risk for progressive or late-onset hearing loss

Children with risk’s factor for progressive or late-onset hearing loss (Table 2) have to undergo an audiologic assessment in a III level centre, once every 6–12 months since the 3rd year of age and then once every year since the 6th year of age:

**Table 2:** Risk factors for progressive or late-onset hearing loss

1.	Positive familiar history for infantile hearing loss
2.	Intrauterine CMV or rubella infections
3.	Syndromes associated with late-onset or progressive hearing loss (Pendred Syndrome, distal renal tubular acidosis (dRTA), Waardenburg syndrome type II, brachio-oto-renal syndrome (BOR), Usher syndrome, Stickler syndrome, CHARGE syndrome, Down syndrome, Turner syndrome, Alport syndrome, exc.
4.	Neurodegenerative disorders, such as Hunter syndrome or sensorimotor neuropathies, such as Friedreich atassia or Charcot-Marie-Tooth syndrome

The personnel in charge of screening’s protocol have to inform the paediatricians about the inclusion of babies in the group of patients at risk for progressive or late-onset hearing loss, during the discharge of the baby from the childbirth centre.

#### Surveillance on children with risk for acquired hearing loss

Children that acquire post-natal infections associated with sensorineural hearing loss (e.g. bacterical meningitis) or suffer for cranial trauma (with loss of consciousness and/or cranial fractures) or sustain chemotherapy (e.g. with cisplatinum) or treatments with ototossic drugs (e.g. aminoglicosidis), have to undergo an audiological evaluation. Further, an audiologic assessment has to be executed in every cases in which the family or the educators suspect an hearing impairment or in every case of a delay in speech development (Table 3).

**Table 3:** Risk for acquired hearing loss

1.	Positive cultural examination for post congenital infections, viral and bacterical meningitis^a^ included
2.	Head trauma, in particularly with skull base’s or temporal bone’s fractures that require hospitalization
3.	Chemotherapy or ototoxic drugs administration^a^
4.	Educator’s concerning on hearing, verbal perception, language development or development delays

^a^in those cases a follow up protocol is needed

Further all the newborns admitted to an hospital during the first months of life, in whom occur hyperbilirubinemia that require exanguino-transfusion, sepsis with positive coultural examination, bacterial meningitis, since those conditions are risk’s factors for hearing loss, must undergo a new screening with TEOAE and AABR within the discharge.

### Role of the family paediatrician

The paediatrician has a key role in the supervision of the execution of screening’s procedure, in the execution of an audiologic follow-up and in the surveillance of children at risk of progressive or late-onset hearing loss (see Table 2); further, paediatricians have to identify the children with risk’s factors for acquired hearing impairment (see Table 3).

The paediatrician has also to verify the correct execution of the screening; and it has to forward children born abroad or in regions in which UNHS is not executed to the responsible audiologic centre; it has to supervise the newborns resulted *refer* at UNHS till the II level retest and oversee on the children with risk’s factors for progressive or late-onset hearing loss, including babies with *refer* result at TEOAE and *pass* at AABR, or those with risk’s factor for acquired hearing loss. The paediatrician, then, must verify that the CMV research in urine with PCR has been executed in all the babies resulted *refer* at screening with TEOAE measurement (see Figs. 1 and 2). Finally, in case of children resulted *refer* at screening’s protocol, the general paediatrician has to verify that the audiological evaluation and diagnosis is carried out in the proper time (within 3 months from birth).Moreover in the new protocol for UNHS in Tuscany region is suggested that the paediatrician execute a constant audiological surveillance with specifically developed surveys. These surveys have been developed with the collaboration of the family paediatricians of the Tuscany region and are aimed to identify red flags of hearing loss. Beside having a key role in identifying cases with acquired, progressive or late-onset hearing loss, the family pediatrician has a key role in the identification of false negative cases (Table 4).

**Table 4:** Pediatrician’s role

1.	Supervise the correct execution of the screening
2.	Forward in the audiologic center for an audiologic evaluation, the babies born in other regions or countries, in which the NHS is not executed
3.	Supervise the *refer* children, till the execution of the II level test
4.	Supervise on babies with risk’s factors for late-onset or progressive hearing loss, including those with *refer* TEOAE and *pass* ABR
5.	Supervise on babies with risk’s factors for acquired hearing loss
6.	Verify cases that CMV research in urine by PCR had been executed in all the *refer* cases
7.	Audiological surveillance with BOEL test and submission of health’s balance questionnaire

## Discussion

UNHS represents the only way to identify children with a congenital moderate-to-profound hearing loss. Thanks to advances in technology both in TEOAE and AABR measurements, UNHS became a very accurate procedure. Relaying on the last generation technologies and on specifically trained personnel, the sensitivity and the specificity of NHS programmes are very high. To date homogeneous data on the sensitivity of UNHS programs are not available, while the reported specificity is about 97–98%.

Measuring both TEOAE and AABR false positive cases are strongly decreased. The possibility of false negative, indeed, is a critical issue since it may lead to a delayed diagnosis of hearing impairment, with consequences on rehabilitation results in terms of hearing abilities and linguistic and communicative skills [4].

After the audiologic assessment of *refer* newborns, the person in charge of the screening procedure should be informed about the audiological diagnosis.

Children with progressive or late-onset hearing loss are not identified by the UNHS tests; the chance to identify them is represented by the programs of long term audiological follow-up and surveillance. Progressive or late onset hearing loss may be related to many causes (genetic predisposition, infections, drugs, exc.) and they represent a large proportion of hearing impairment in children (20–30%), even if reliable international data on the subject are not available [5, 6].

In the same way, babies with acquired hearing loss (e.g. due to bacterial meningitis) are not identified by the screening, and those cases can be precociously identified by the audiological surveillance’s programs [5, 6].

The JCIH in 2007 Position Statement, identified the problems related with progressive, late-onset and acquired hearing loss, and it defined the risk’s factors in which is recommended an audiologic follow-up in the first years after the birth [1]. The classification of risk’s factors reported in the JCIH Position Statement [7] is complex: plenty of factors are reported together, with different risk’s indexes (low and high risk). Moreover, Italian and American health systems are so different that the classification had got to be necessarily adapted to the Italian health reality.

For some of the risk’s factors reported in the 2007 JCIH position statement, the real possibility to cause progressive or late onset hearing loss has not been determined clearly in scientific literature yet, or it is considered not-significant or very weak (e.g. congenital siphilidis or herpes simplex). In fact, the most recent studies in scientific literature tend to simplify the list of risk’s factors for progressive or late-onset hearing loss; it also facilitate the execution of a more selective and effective follow-up protocol [5, 6, 8].

Moreover, in 2007 JCIH statement, risk’s factors are reported in the same list, those present at birth (e.g. CMV congenital infection) and those occurred in the perinatal period which can lead to progressive or late-onset hearing loss (e.g. prematurity associated conditions). Acquired conditions, indeed, should be considered independently, since they require a different surveillance’s protocols.

An audiologic evaluation within the 24 and the 30 months for children with low risk’s condition for progressive or late-onset hearing loss, as suggested by the JCIH (2007) may be excessive, difficult and useless, causing an excessive work loading for the Sanitary System with a not clear cost/benefit rate. We believe that in those cases a surveillance by the general paediatrician could be extremely useful, with the submission of specifically developed questionnaires to parents. Babies identified in this surveillance protocol and children with speech delay must be addressed by the paediatrician to the responsible audiologic centre, for the deepening of the evaluation with tests appropriated for the age.

Due to these critical issues and after a revision of the recent literature on the subject, we considered to modify and simplify the list of risk’s factors, classifying them into two different distinct tables: risk’s factors for progressive and late-onset hearing loss (Table 2) and acquired condition that may lead to acquired hearing loss (Table 3). The enlisted factors are some of those indicated as high-risk’s factors by JCIH in the 2007 Position Statement. Risk’s factors reported as low-risk by JCIH has been deleted, since not clearly related with an increased risk of progressive or late-onset hearing loss in the most recent epidemiological studies [1].

Another issue related to UNHS program is represented by the possibility to miss some newborns with light or mild hearing deficits, limited to high frequencies. In fact, tests used for the I and II level screening (TOEAE and AABR), even if very reliable, cannot identify such deficits, for technical limits of the tests.

Another possible cause of missed identification of an hearing impairment is related with the auditory neuropathy (AN); in fact a screening executed with the measurement of TEOAE only is not capable to identify the affected cases. There are some cases in which newborns have an higher risk to develop AN. The JCIH statement of 2007 did not specifically enlist the risk’s factors for AN. In the new Tuscany region’s guidelines, such risk’s factors have been identified, taking inspiration from the audiological risk’s factors reported in the Position Statement of JCIH, with some modifications and simplifications (Table 1). Newborns that present risk’s factors reported in Table 1 has to undergo the screening with TOAEO and AABR measurement. Anyway, we should consider that from 20 to 40% of AN cases may occur in newborns/children without risk’s factor reported in Table 1, as reported in some recent literature articles [9]. Those cases unavoidably escape from the screening executed with TEOAE only, so for the early identification of cases with AN it is crucial an active audiological surveillance, in which the general paediatrician has a key role in this.

We believe that for these mentioned reasons a program of audiological follow-up and surveillance is crucial. In Italy each child is addressed to a general paediatrician, so the it plays a key role in the audiological follow-up and surveillance. In Tuscany region’s screening protocol, the role of the general paediatrician is underlined and it is suggested that the paediatrician execute an audiological surveillance during the periodical controls with a specifically developed survey, reported in Additional file 1. To this regard, in 2010 all the general paediatrician of Tuscany region participated to mandatory training courses in paediatric audiology and during these courses has been discussed the development of a specific audiologic survey. This survey has been developed then, and it has been included in the new guidelines and approved by the Regional Sanitary Council in August 2016. These questionnaires investigate about the hearing and communicative behaviour of children and about the possible presence of hearing impairments. All the children must undergo the survey, independently from the result of NHS or from the presence of risk’s factor for hearing impairment, in order to identify the cases of acquired or progressive or late-onset hearing loss, or missed cases at the screening’s procedures.

Another considerable issue in the audiological newborn screening protocols and in the early identification and treatment of infantile hearing impairments is represented by the congenital CMV infection (cCMV) [10].

Nowadays cCMV infection is considered the first non genetic cause of infantile deafness and it is an important reason for many infantile neurodevelopmental disorders; anyway, since most of newborns with cCMV is symptomless at birth and since a screening protocol for cCMV does not exist yet, the actual impact and the real frequency of this important infection are not yet precisely defined.

For a certain diagnosis of cCMV a strict temporal window is available; it is limited at the first 2 or 3 weeks after birth. After this period is difficult to discriminate from a congenital and an acquired infection. Those diagnostic issues, together with the lack of symptoms at birth in most of newborns with cCMV, are responsible of many cases of misdiagnosed congenital CMV infection [11]. So, the addition of CMV testing would help a NHS program to attain an early audiological diagnosis and to programme an accurate follow-up, as also recently reported by Diener and colleagues [12].

Further, is it well known that is extremely important to identify a cCMV as soon as possible. Indeed, many cases of cCMV related deafness are progressive or late-onset and so, an early identification of cCMV, makes possible the execution of an audiological follow up. Further, an early diagnosis, within the first month from the birth, make possible to execute, in selected cases, an antiviral drug treatment in an optimal temporal window [13].

As far as we know, the NHS protocol of the Tuscany regions is the first to propose and implement a screening for CMV congenital infection in all the newborns refer at TEOAE measurement, by the research of CMV genome by PCR on urine. This procedure permits an early diagnosis of an eventual cCMV; further it allow to avoid false positive results and it resolve the diagnostic doubts on the possibility of a post-natal infection; furthermore it is important for making possible to set up an audiologic follow up in affected babies and for guarantee the chance to submit the affected babies to the recent therapeutic strategies with antiviral drugs.

Williams et al. [14] have recently released a study on the feasibility of an early diagnostic protocol for cCMV, and on the acceptability of a screening for cCMV, limited in babies resulted *refer* at NHS. The study concluded that the screening for cCMV, executed on saliva of babies resulted *refer* at NHS is feasible and well accepted from the families and, above all, it permits to identify cases that may benefit from antiviral drugs. Recently our group published a study on the incidence of cCMV in preterm and small for gestational age (SGA) babies, hospitalized in Neonatology Operative Unit of Azienda Ospedaliero Universitaria of Pisa (AOUP) that have been submitted at screening protocol. An high incidence of cCMV has been found in preterm (3,03%) and SGA (3,7%) babies. The babies resulted positive for cCMV resulted also *refer* at TOAE measurement in the 25% of cases and at ABR meas in 16,7%. According on this study it seems to exist an important association between cCMV and prematurity or SGA condition at birth [11].

## Conclusions

The execution of UNHS is the only way to identify newborns with congenital moderate to profound hearing loss. Progressive or late onset hearing loss represents a relatively large proportion of childhood hearing impairments and may be not identified by the screening, as well as acquired hearing loss, light-mild hearing loss or impairments limited to the high frequencies and hearing loss related to AN or neural deafness. To early identify those cases an audiological follow-up program is necessary, as well as an audiological surveillance protocol.

The new NHS protocol in Tuscany region considered all the mentioned issues, as well as it underscores the role of the general paediatrician in supervising the screening execution and the audiological evaluation, and the audiological follow-up and surveillance.

Finally, in consideration of the importance of cCMV as a cause of hearing loss in childhood, in Tuscany region all the newborns resulted *refer* at TEOAE measurement are early investigated for CMV congenital infection by the research of CMV DNA in urine by PCR. In Table 5 the main features of the protocol are summarised.

**Table 5:** Tuscany region’s screening protocol key points

1.	Identification of risk factors for AN and execution of TEOAE and ABR measurements in children with risk’s factors
2.	Identification of risk factors for progressive or late onset hearing loss. Audiological assessment in a III level centre, once every 6–12 months since the 3rd year of age and then once every year since the 6th year of age, in children with risk for progressive or late-onset hearing loss
3.	Identification of risk factors for acquired hearing loss
4.	Key role of the paediatrician in the supervision on the execution of screening’s procedure, in the audiologic follow-up, in the surveillance of children at risk of progressive or late-onset hearing loss and in the identification of children with risk’s factors for acquired hearing impairment
5.	Screening for CMV congenital infection in all the children resulted *refer* at TEOAE measurement, by the research of CMV genome by PCR on urine, within the 15th day after birth

## Additional file

Additional file 1: Survey for audiologic survelliance (DOC 25 kb)

## Abbreviations

AABR: Automatic auditory brainstem responses
ABR: Clinical auditory brainstem responses
AN: Auditory neuropathy
AOUP: Azienda ospedaliero universitaria of Pisa (universital ospital of Pisa)
cCMV: Congenital citomegalovirus infection
CI: Cochlear implant
CMV: Citomegalovirus
DPOAE: Distortion Produced Oto-Acoustic Emissions
ENT: Ear, nose and throat
HA: Hearing aids
HTA: Italian Health Technology Assessment
JCIH: Joint Committee on Infant Hearing
LEA: “livelli essenziali di assistenza”
NHS: Newborn hearing screening
NICU: Neonatal intensive care unit
PCR: Polymerase Chain Reaction
SGA: Small for gestational age
TEOAE: Transient Evoked Oto-Acoustic Emissions
UNHS: Universal newborn hearing screening

## References

[1] American Academy of Pediatrics JCoIH (2007). Year 2007 position statement: Principles and guidelines for early hearing detection and intervention programs. Pediatrics.

[2] Ghirri P, Liumbruno A, Lunardi S (2011). Universal neonatal audiological screening: experience of the University Hospital of Pisa. Ital J Pediatr.

[3] Berrettini S, Arslan E, Baggiani A, Burdo S, Cassandro E, Cuda D, Filipo R, Rossi PG, Mancini P, Martini A, Quaranta A, Quaranta N, Turchetti G, Forli F (2011). Analysis of the impact of professional involvement in evidence generation for the HTA Process, subproject “cochlear implants”: methodology, results and recommendations. Acta Otorhinolaryngol Ital.

[4] Wroblewska-Seniuk KE, Dabrowski P, Szyfter W, Mazela J (2017). Universal newborn hearing screening: methods and results, obstacles, and benefits. Pediatr Res.

[5] Beswick R, Driscoll C, Kei J (2012). Monitoring for postnatal hearing loss using risk factors: a systematic literature review. Ear Hear.

[6] Wood SA, Davis AC, Sutton GJ (2013). Effectiveness of targeted surveillance to identify moderate to profound permanent childhood hearing impairment in babies with risk factors who pass newborn screening. Int J Audiol.

[7] Watkin PM, Baldwin M (2011). Identifying deafness in early childhood: requirements after the newborn hearing screen. Arch Dis Child.

[8] Vos B, Senterre C, Lagasse R, Levêque A, SurdiScreen Group (2015). Newborn hearing screening programme in Belgium: a consensus recommendation on risk factors. BMC Pediatr.

[9] Boudewyns A, Declau F, van den Ende J, Hofkens A, Dirckx S, Van de Heyning P (2016). Auditory neuropathy spectrum disorder (ANSD) in referrals from neonatal hearing screening at a well-baby clinic. Eur J Pediatr.

[10] Goderis J, De Leenheer E, Smets K, Van Hoecke H, Keymeulen A, Dhooge I (2014). Hearing loss and congenital CMV infection: a systematic review. Pediatrics.

[11] Lorenzoni F, Lunardi S, Liumbruno A (2014). Neonatal screening for congenital cytomegalovirus infection in preterm and small for gestational age infants. J Matern Fetal Neonatal Med.

[12] Diener ML, Zick CD, McVicar SB, Boettger J, Park AH. Outcomes From a Hearing-Targeted Cytomegalovirus Screening Program. Pediatrics*.* 2017;139(2). 10.1542/peds.2016-0789 28119425

[13] Kimberlin DW, Jester PM, Sanchez PJ (2015). Valganciclovir for symptomatic congenital cytomegalovirus disease. N Engl J Med.

[14] Williams EJ, Kadambari S, Berrington JE (2014). Feasibility and acceptability of targeted screening for congenital CMV-related hearing loss. Arch Dis Child Fetal Neonatal Ed.

