# Supplementary material for: Newborn hearing screening protocol in tuscany region
Source: Ital J Pediatr. 2017 Sep 20;43:82. doi: 10.1186/s13052-017-0397-1 (PMC5607492; doi:10.1186/s13052-017-0397-1)
Supplement: Additional file 1: — Survey for audiologic survelliance (DOC 25 kb) [file 13052_2017_397_MOESM1_ESM.doc]

APPENDIX 1

**Survey for Audiologic Survelliance**

From the taken in charge till the 3rd month

 Does the child get scared for loud noises?

 Are you concerned about your child hearing?

At 6 th month

 Does the child react to voices and noises?

 Does the child smile in reaction to voices?

 Does he pay attention when you speak toward him?

 Does he produce noises or sounds which contain AA, EE, GA, GE vowels?

 Does he like listen to the music?

 Does he like the sound of a toys or a carillon?

 Do you think your child has a normal hearing?

At 8 th month

Do the BOEL TEST

 Does the child produce syllables in series (ma-ma, ba-ba, da-da)?

 Does he orientate himself correctly if a sound comes laterally or from behind?

 Do you have any doubt on the hearing of your child?

At 12 th month

 Does the child understand his name?

 Does he react at the telephone ring or at the door bell?

 Does he localize sounds?

 Does he seems to understand words or simple sentences?

 Does he continue to produce series of syllables, with an higher variability then

before, similar to complete words?

 Do you have any doubt on the hearing of your child?

At 18 th month

 Does the child produce ten words, even if not correctly, and does he start to put them

together?

 Does he react if you call him from another room?

 Do you have any doubt on the hearing of your child?

At the 3 rd year

 Does the child speak and listen to the telephone?

 Does his vocabulary constantly expand?

 Does he produce simple sentences, even if not correctly?

 Does he listen gladly to stories or tales?

 Do you have any doubt on the hearing of your child?
